# Supplementary material for: A Likelihood Approach for Real-Time Calibration of Stochastic Compartmental Epidemic Models
Source: PLoS Comput Biol. 2017 Jan 17;13(1):e1005257. doi: 10.1371/journal.pcbi.1005257 (PMC5240920; doi:10.1371/journal.pcbi.1005257)
Supplement: S1 File — (TAR.GZ) [file pcbi.1005257.s014.tar.gz › HSPH_Online-SI-Revision/Additions/EnKF08/n100-extreme-SEITR/n100-extreme-SEITR_EnKF2_table-4.pdf]

1	newly 3 weeks spec	13598.	696.57	{0., 1001.}	97.1875	3.26462	newly 3 weeks cumul	31455.	12175.9	{818., 11568.}	84.4958	0.804228	newly 5 weeks spec	12085.	162.22	{0., 507.}	98.6577	3.64149	newly 5 weeks cumul	57301.	12661.1	{839., 11568.}	83.4022	0.989103	newly 55 weeks cumul	84027.	13063.8	{841., 12884.}	84.8085	1.10355
2	newly 3 weeks spec	17235.	1278.12	{271., 2795.}	92.5842	1.36506	newly 3 weeks cumul	29177.	23898.6	{8466., 68601.}	73.2776	0.407967	newly 5 weeks spec	17453.	200.12	{6., 218.}	98.8534	2.65091	newly 5 weeks cumul	67910.	24562.8	{8598., 68921.}	66.0353	0.615534	newly 62 weeks cumul	97717.	24764.6	{8613., 68927.}	74.6569	0.761608
3	newly 3 weeks spec	14424.	7148.19	{966., 19303.}	62.0254	0.577221	newly 3 weeks cumul	24222.	49187.8	{10355., 116796.}	132.249	0.34383	newly 5 weeks spec	18266.	1828.06	{69., 3900.}	89.992	1.36574	newly 5 weeks cumul	61841.	54520.4	{12016., 118959.}	62.9813	0.36218	newly 64 weeks cumul	97783.	57138.5	{12433., 120123.}	52.2818	0.410599
4	newly 3 weeks spec	13822.	1209.86	{255., 1833.}	91.2469	1.31591	newly 3 weeks cumul	25848.	21665.4	{6655., 58338.}	70.1479	0.39766	newly 5 weeks spec	16022.	192.05	{4., 229.}	98.8013	2.63623	newly 5 weeks cumul	59339.	22303.	{6733., 59101.}	66.3522	0.605768	newly 62 weeks cumul	94062.	22479.9	{6940., 59157.}	76.1009	0.783841
5	newly 3 weeks spec	22578.	5310.29	{901., 9566.}	76.5768	0.797598	newly 3 weeks cumul	36477.	40925.9	{6512., 97218.}	71.9382	0.332247	newly 5 weeks spec	16920.	1702.81	{126., 3866.}	90.2298	1.40337	newly 5 weeks cumul	77798.	45434.9	{8788., 108967.}	57.6555	0.437478	newly 64 weeks cumul	97923.	48490.8	{10582., 113069.}	56.7057	0.466725
6	newly 3 weeks spec	20143.	3004.58	{1., 8405.}	85.0838	2.10302	newly 3 weeks cumul	34123.	38620.8	{6985., 89721.}	72.9315	0.371875	newly 5 weeks spec	17405.	1095.35	{0., 4218.}	93.7067	3.08852	newly 5 weeks cumul	74866.	41410.2	{7229., 105913.}	58.1491	0.505231	newly 63 weeks cumul	93450.	43969.8	{7274., 114431.}	59.7259	0.557188
7	newly 3 weeks spec	12108.	1089.38	{0., 1749.}	94.9349	3.12586	newly 3 weeks cumul	28975.	10438.3	{1870., 10602.}	81.5944	0.777447	newly 5 weeks spec	11332.	644.67	{0., 1422.}	94.9115	3.53827	newly 5 weeks cumul	52923.	11832.8	{1950., 11647.}	84.6655	0.965266	newly 55 weeks cumul	88800.	17476.7	{1963., 87501.}	82.2964	1.09866
8	newly 3 weeks spec	16980.	1134.23	{240., 1729.}	93.3202	1.37229	newly 3 weeks cumul	32632.	16793.7	{7381., 39398.}	63.6028	0.450362	newly 5 weeks spec	14766.	337.54	{4., 285.}	97.7141	2.47043	newly 5 weeks cumul	65812.	17684.6	{7532., 39521.}	73.3414	0.680257	newly 62 weeks cumul	97109.	18422.2	{8464., 39522.}	81.0294	0.823578
9	newly 3 weeks spec	23304.	6841.15	{883., 20658.}	71.3483	0.75484	newly 3 weeks cumul	35304.	45791.5	{9607., 103573.}	82.328	0.343773	newly 5 weeks spec	17411.	1841.69	{70., 3816.}	89.4223	1.36988	newly 5 weeks cumul	78660.	51039.2	{11177., 123006.}	58.6476	0.439924	newly 64 weeks cumul	98833.	53997.7	{11760., 123576.}	55.125	0.455634
10	newly 3 weeks spec	12030.	2012.91	{1., 1626.}	97.6807	2.13403	newly 3 weeks cumul	25011.	6393.66	{1032., 18305.}	90.8324	1.09858	newly 5 weeks spec	13614.	874.06	{0., 279.}	93.8734	2.91552	newly 5 weeks cumul	52825.	8780.28	{1032., 24429.}	90.0295	1.34996	newly 60 weeks cumul	95503.	9988.29	{1047., 24473.}	89.5414	1.56023
11	newly 3 weeks spec	13896.	2272.71	{166., 13549.}	85.7875	1.4064	newly 3 weeks cumul	26326.	11809.4	{1340., 68660.}	98.917	0.952791	newly 5 weeks spec	14675.	518.21	{18., 1821.}	96.4688	2.05793	newly 5 weeks cumul	57151.	13380.5	{1664., 75854.}	87.3799	1.15837	newly 59 weeks cumul	97210.	14527.9	{1780., 76908.}	86.5279	1.32098
12	newly 3 weeks spec	16108.	2237.76	{0., 1727.}	94.8051	2.62834	newly 3 weeks cumul	33166.	7382.79	{563., 33840.}	88.0752	1.25462	newly 5 weeks spec	14159.	948.2	{0., 329.}	93.3032	3.14429	newly 5 weeks cumul	64453.	10064.7	{563., 33844.}	87.35	1.47096	newly 60 weeks cumul	95054.	11170.8	{563., 59760.}	88.248	1.60839
13	newly 3 weeks spec	15592.	800.41	{0., 788.}	94.8665	2.70372	newly 3 weeks cumul	30732.	8486.72	{698., 19715.}	76.1409	0.845707	newly 5 weeks spec	14704.	453.28	{0., 188.}	96.9173	3.49015	newly 5 weeks cumul	62511.	9560.55	{759., 19731.}	85.2704	1.11816	newly 61 weeks cumul	96828.	10309.9	{774., 19731.}	89.3523	1.29491
14	newly 3 weeks spec	10081.	1268.88	{58., 1093.}	93.73	1.53462	newly 3 weeks cumul	23444.	22089.6	{2114., 73319.}	130.76	0.750946	newly 5 weeks spec	11642.	285.97	{0., 173.}	97.5436	2.92894	newly 5 weeks cumul	46832.	22946.9	{2126., 73322.}	103.56	0.962497	newly 54 weeks cumul	89533.	23224.6	{2126., 76800.}	90.607	1.17238
15	newly 3 weeks spec	14236.	654.05	{0., 870.}	96.4456	3.45984	newly 3 weeks cumul	30841.	10149.3	{99., 9824.}	85.0193	1.01278	newly 5 weeks spec	13230.	124.07	{0., 406.}	99.0622	3.85472	newly 5 weeks cumul	59486.	10537.4	{99., 9824.}	85.9809	1.22717	newly 55 weeks cumul	91749.	10830.5	{99., 9824.}	88.3029	1.36471
16	newly 3 weeks spec	11254.	1209.05	{23., 3976.}	91.6998	1.77368	newly 3 weeks cumul	24092.	6155.75	{239., 17685.}	90.0193	1.18949	newly 5 weeks spec	12953.	377.63	{0., 635.}	97.0846	2.62957	newly 5 weeks cumul	50447.	7195.62	{284., 22250.}	89.0265	1.4216	newly 58 weeks cumul	87287.	7946.14	{363., 25831.}	91.5097	1.61627
17	newly 3 weeks spec	15615.	5334.94	{906., 9942.}	71.4979	0.661404	newly 3 weeks cumul	24517.	42099.3	{13272., 112352.}	100.84	0.273773	newly 5 weeks spec	20574.	1415.9	{69., 3486.}	93.118	1.57603	newly 5 weeks cumul	68260.	46093.3	{14788., 120133.}	60.036	0.384989	newly 64 weeks cumul	92364.	48565.1	{14863., 120670.}	57.6441	0.432713
18	newly 3 weeks spec	19007.	2856.5	{1., 8447.}	85.0674	2.04628	newly 3 weeks cumul	31170.	41472.3	{6891., 89737.}	77.2283	0.3098	newly 5 weeks spec	17189.	866.19	{0., 3112.}	94.9608	3.1722	newly 5 weeks cumul	70235.	43816.9	{7127., 106267.}	58.2882	0.432236	newly 63 weeks cumul	98793.	45557.5	{7172., 114998.}	61.0033	0.5082
19	newly 3 weeks spec	12889.	1533.4	{23., 5993.}	91.4927	1.49265	newly 3 weeks cumul	24953.	8748.58	{2211., 40316.}	88.9034	0.856857	newly 5 weeks spec	14542.	530.91	{0., 1603.}	96.3491	2.32398	newly 5 weeks cumul	54787.	10187.3	{2606., 45145.}	86.2481	1.08793	newly 58 weeks cumul	96606.	11040.5	{2699., 46866.}	90.298	1.28751
20	newly 3 weeks spec	19368.	2138.36	{4., 6899.}	88.9593	1.98805	newly 3 weeks cumul	33454.	36096.9	{6538., 73716.}	62.9431	0.303668	newly 5 weeks spec	16864.	731.64	{0., 3340.}	95.6615	3.28448	newly 5 weeks cumul	72041.	37982.9	{6778., 73716.}	57.4818	0.456774	newly 63 weeks cumul	97397.	40286.5	{6793., 73716.}	61.9724	0.534984
21	newly 3 weeks spec	19672.	5826.25	{848., 15277.}	71.8608	0.77808	newly 3 weeks cumul	30439.	47545.2	{12243., 107242.}	90.2475	0.284271	newly 5 weeks spec	18543.	1475.51	{42., 3607.}	92.0428	1.60897	newly 5 weeks cumul	73114.	51826.3	{13675., 107944.}	56.5689	0.374192	newly 64 weeks cumul	98246.	53975.6	{13972., 111912.}	53.5149	0.417969
22	newly 3 weeks spec	14910.	3020.54	{0., 12779.}	93.3345	2.2956	newly 3 weeks cumul	31688.	8806.48	{301., 47061.}	88.265	1.18691	newly 5 weeks spec	14003.	1193.23	{0., 8400.}	91.6924	2.88289	newly 5 weeks cumul	61831.	12219.4	{372., 64609.}	85.7094	1.39276	newly 60 weeks cumul	92206.	13573.7	{410., 81392.}	85.4898	1.52288
23	newly 3 weeks spec	16317.	1130.82	{0., 1244.}	93.0697	2.57924	newly 3 weeks cumul	34910.	8959.95	{1745., 17695.}	77.523	0.823439	newly 5 weeks spec	13260.	586.44	{0., 207.}	95.5774	3.387	newly 5 weeks cumul	65072.	10382.3	{1799., 17702.}	84.1232	1.05662	newly 61 weeks cumul	83725.	11312.	{1802., 17702.}	86.4891	1.15549
24	newly 3 weeks spec	16757.	2242.04	{1., 7138.}	87.9548	1.93322	newly 3 weeks cumul	25641.	29053.8	{5223., 69932.}	72.0933	0.342465	newly 5 weeks spec	20012.	654.35	{0., 1984.}	96.7302	2.98166	newly 5 weeks cumul	69280.	30838.7	{6365., 69932.}	65.9429	0.567844	newly 63 weeks cumul	98228.	32245.1	{7507., 69932.}	70.9037	0.678143
25	newly 3 weeks spec	12372.	1192.36	{16., 756.}	93.6786	1.69939	newly 3 weeks cumul	23919.	7351.29	{93., 3816.}	102.315	1.1937	newly 5 weeks spec	15457.	332.11	{9., 234.}	97.8514	2.40045	newly 5 weeks cumul	55191.	8290.8	{115., 4066.}	94.4118	1.45862	newly 59 weeks cumul	91517.	8948.84	{127., 7898.}	92.6021	1.62113
26	newly 3 weeks spec	12283.	2623.9	{154., 12268.}	84.36	1.26627	newly 3 weeks cumul	28429.	12624.6	{1246., 30331.}	101.062	1.01524	newly 5 weeks spec	11730.	1360.37	{21., 1475.}	90.5123	1.64059	newly 5 weeks cumul	53475.	15846.7	{1614., 55323.}	89.7768	1.13863	newly 57 weeks cumul	80102.	19803.9	{1868., 86132.}	84.148	1.1803
27	newly 3 weeks spec	12409.	2512.21	{126., 10673.}	88.2618	1.35301	newly 3 weeks cumul	28757.	11742.1	{1072., 24351.}	101.473	1.11547	newly 5 weeks spec	11908.	980.77	{20., 1572.}	92.4376	1.68045	newly 5 weeks cumul	53686.	14236.9	{1416., 48465.}	91.5835	1.2297	newly 57 weeks cumul	90968.	18818.4	{1848., 86237.}	83.7234	1.1761
28	newly 3 weeks spec	16959.	7057.09	{809., 20284.}	64.7784	0.658113	newly 3 weeks cumul	28083.	45791.9	{3913., 99667.}	109.941	0.416503	newly 5 weeks spec	18063.	1943.38	{79., 3777.}	89.2411	1.34185	newly 5 weeks cumul	68202.	51308.8	{5030., 117061.}	61.4014	0.467364	newly 64 weeks cumul	88415.	54231.6	{6390., 117358.}	54.949	0.478372
29	newly 3 weeks spec	12914.	583.27	{0., 517.}	95.6549	2.40912	newly 3 weeks cumul	24569.	6367.28	{1127., 15316.}	79.3562	0.853338	newly 5 weeks spec	15388.	207.56	{0., 102.}	98.6512	3.30626	newly 5 weeks cumul	56007.	6912.74	{1261., 18936.}	88.2416	1.17364	newly 61 weeks cumul	96532.	7230.4	{1284., 18936.}	92.5098	1.39899
30	newly 3 weeks spec	13125.	5588.29	{842., 15148.}	68.0543	0.633236	newly 3 weeks cumul	20142.	40306.7	{5829., 96917.}	134.425	0.364285	newly 5 weeks spec	19690.	1401.5	{75., 3540.}	92.8822	1.53987	newly 5 weeks cumul	60177.	44387.9	{6679., 108689.}	64.5069	0.441343	newly 64 weeks cumul	98710.	46566.6	{6971., 112811.}	58.7213	0.528115
31	newly 3 weeks spec	10370.	739.08	{9., 710.}	95.3851	1.60558	newly 3 weeks cumul	26082.	10796.6	{2907., 13022.}	95.5866	0.799632	newly 5 weeks spec	9931.	217.01	{0., 216.}	97.9655	2.56291	newly 5 weeks cumul	46868.	11427.7	{3088., 13039.}	91.4006	0.992026	newly 54 weeks cumul	76589.	11643.6	{3088., 13039.}	90.063	1.17144
32	newly 3 weeks spec	12614.	1564.51	{0., 1362.}	95.7876	2.30052	newly 3 weeks cumul	26998.	5626.96	{719., 13696.}	88.33	1.10767	newly 5 weeks spec	13001.	691.76	{0., 269.}	95.0405	3.00368	newly 5 weeks cumul	54036.	7486.65	{745., 16405.}	89.5872	1.3467	newly 60 weeks cumul	93822.	8370.57	{759., 17276.}	91.095	1.55512
33	newly 3 weeks spec	20264.	6707.66	{822., 20443.}	69.7124	0.713364	newly 3 weeks cumul	37971.	48641.4	{11636., 102040.}	81.4082	0.342515	newly 5 weeks spec	15166.	1647.49	{51., 3669.}	89.137	1.38605	newly 5 weeks cumul	73450.	53503.5	{12153., 120521.}	57.1157	0.398516	newly 64 weeks cumul	96763.	55677.	{12235., 121111.}	53.6084	0.434561
34	newly 3 weeks spec	15602.	1615.96	{0., 2653.}	93.1818	2.38251	newly 3 weeks cumul	34185.	7525.96	{1029., 25647.}	84.6082	1.12607	newly 5 weeks spec	13645.	908.48	{0., 304.}	94.0605	3.17556	newly 5 weeks cumul	63916.	9727.13	{1096., 38752.}	86.7942	1.33844	newly 60 weeks cumul	89965.	11329.8	{1098., 38762.}	87.5605	1.4563
35	newly 3 weeks spec	18683.	6580.54	{893., 15336.}	69.9168	0.732586	newly 3 weeks cumul	29482.	43609.8	{10806., 117904.}	93.0672	0.329576	newly 5 weeks spec	19217.	2088.14	{70., 4633.}	89.1339	1.43468	newly 5 weeks cumul	73185.	49268.4	{11880., 121287.}	60.6369	0.43442	newly 64 weeks cumul	94833.	53071.7	{12198., 121499.}	57.0596	0.463234
36	newly 3 weeks spec	11929.	2477.91	{1., 2107.}	98.7976	2.10312	newly 3 weeks cumul	21930.	7066.4	{998., 8038.}	95.5082	1.02236	newly 5 weeks spec	15024.	906.21	{0., 3494.}	93.9683	2.8945	newly 5 weeks cumul	52068.	9715.96	{998., 11199.}	91.5839	1.30705	newly 60 weeks cumul	97587.	11174.2	{998., 40524.}	89.0549	1.51536
37	newly 3 weeks spec	15965.	606.45	{0., 480.}	96.2014	2.54779	newly 3 weeks cumul	25084.	9916.17	{874., 19800.}	80.5252	0.780703	newly 5 weeks spec	18951.	132.82	{0., 82.}	99.2991	3.54064	newly 5 weeks cumul	65780.	10327.2	{969., 19815.}	86.68	1.14264	newly 61 weeks cumul	98650.	10474.3	{1021., 19815.}	89.3824	1.305
38	newly 3 weeks spec	14686.	1347.64	{15., 5449.}	91.6263	1.84906	newly 3 weeks cumul	31691.	8235.45	{123., 37565.}	86.0883	1.30341	newly 5 weeks spec	13535.	452.16	{0., 1348.}	96.6593	2.49549	newly 5 weeks cumul	60924.	9457.26	{151., 41629.}	87.0186	1.48856	newly 58 weeks cumul	87770.	10497.2	{195., 42985.}	89.0211	1.58309
39	newly 3 weeks spec	14352.	2290.36	{0., 8382.}	92.6596	2.33604	newly 3 weeks cumul	28679.	6445.47	{960., 24000.}	85.049	1.12449	newly 5 weeks spec	15042.	1370.07	{0., 6887.}	90.9414	3.05504	newly 5 weeks cumul	60279.	9815.73	{960., 35607.}	86.4359	1.37127	newly 60 weeks cumul	93593.	12073.3	{960., 50177.}	87.1002	1.52591
40	newly 3 weeks spec	14498.	393.28	{0., 499.}	97.2873	2.37145	newly 3 weeks cumul	31327.	6631.65	{1223., 12475.}	79.8494	0.865076	newly 5 weeks spec	13194.	91.76	{0., 98.}	99.3045	3.27517	newly 5 weeks cumul	59514.	6905.58	{1445., 13830.}	88.3967	1.12138	newly 61 weeks cumul	94658.	7042.9	{1463., 14302.}	92.5596	1.31313
41	newly 3 weeks spec	13435.	5918.39	{963., 10080.}	62.1162	0.520072	newly 3 weeks cumul	19619.	44664.	{11745., 120349.}	145.79	0.338745	newly 5 weeks spec	23349.	1530.17	{111., 3531.}	93.4465	1.49131	newly 5 weeks cumul	66595.	49079.7	{12260., 122582.}	56.9195	0.35922	newly 64 weeks cumul	95882.	51538.9	{12332., 122759.}	55.1138	0.422236
42	newly 3 weeks spec	10315.	894.01	{0., 1000.}	94.8067	2.97744	newly 3 weeks cumul	25161.	12337.2	{2256., 12445.}	82.43	0.674734	newly 5 weeks spec	10541.	304.35	{0., 653.}	97.1127	3.43276	newly 5 weeks cumul	46907.	13120.9	{2682., 12447.}	83.2505	0.866187	newly 55 weeks cumul	87719.	14288.2	{2797., 12520.}	84.9722	1.06063
43	newly 3 weeks spec	12045.	1782.41	{2., 716.}	96.8617	2.03619	newly 3 weeks cumul	24152.	6431.66	{956., 7610.}	92.4855	1.04952	newly 5 weeks spec	14237.	586.56	{0., 222.}	95.88	2.83192	newly 5 weeks cumul	52694.	8172.44	{956., 7760.}	91.0714	1.31399	newly 60 weeks cumul	95113.	8927.75	{956., 24117.}	90.8133	1.52437
44	newly 3 weeks spec	16196.	2841.93	{1., 9605.}	92.1907	2.14176	newly 3 weeks cumul	32797.	8829.33	{1013., 43996.}	84.7696	1.07857	newly 5 weeks spec	14170.	1240.19	{0., 7072.}	91.5893	2.88212	newly 5 weeks cumul	64055.	12233.9	{1016., 61160.}	84.2096	1.28786	newly 60 weeks cumul	95957.	13677.8	{1016., 80081.}	85.7459	1.43035
45	newly 3 weeks spec	16795.	1194.24	{222., 1854.}	92.8893	1.45102	newly 3 weeks cumul	31747.	21652.9	{7911., 61528.}	70.4234	0.478044	newly 5 weeks spec	14996.	191.62	{2., 209.}	98.7222	2.73414	newly 5 weeks cumul	65154.	22287.3	{8731., 69112.}	70.6689	0.686883	newly 62 weeks cumul	96985.	22462.7	{8748., 69436.}	78.0544	0.843942
46	newly 3 weeks spec	15111.	6175.6	{1020., 14758.}	67.4675	0.612484	newly 3 weeks cumul	25292.	44596.	{10085., 102089.}	108.302	0.309208	newly 5 weeks spec	17991.	1880.77	{115., 4687.}	89.5461	1.42195	newly 5 weeks cumul	63693.	49738.3	{10732., 114623.}	58.4858	0.359651	newly 64 weeks cumul	97957.	53107.9	{11260., 121700.}	54.5238	0.423484
47	newly 3 weeks spec	13356.	1606.74	{120., 2929.}	90.3759	1.58892	newly 3 weeks cumul	27095.	8393.15	{1256., 9880.}	96.6532	1.09483	newly 5 weeks spec	14547.	370.71	{10., 1528.}	97.4516	2.24596	newly 5 weeks cumul	57180.	9537.27	{1400., 13578.}	90.0835	1.31455	newly 59 weeks cumul	92154.	10013.5	{1448., 17407.}	90.0403	1.46988
48	newly 3 weeks spec	14508.	1266.	{326., 1870.}	91.2738	1.27345	newly 3 weeks cumul	25267.	19792.8	{5924., 63345.}	76.3682	0.416756	newly 5 weeks spec	17877.	221.24	{9., 273.}	98.7624	2.50567	newly 5 weeks cumul	62499.	20486.9	{6694., 65767.}	73.61	0.68103	newly 62 weeks cumul	95356.	20802.3	{7016., 65869.}	78.8052	0.837022
49	newly 3 weeks spec	15110.	2195.54	{0., 1415.}	94.8715	2.3593	newly 3 weeks cumul	34829.	6574.45	{983., 19135.}	86.7758	1.1501	newly 5 weeks spec	12499.	877.13	{0., 1215.}	93.43	2.98251	newly 5 weeks cumul	62570.	9052.99	{983., 21515.}	88.0846	1.34599	newly 60 weeks cumul	88106.	10178.4	{983., 22165.}	88.4476	1.4621
50	newly 3 weeks spec	13796.	522.25	{0., 487.}	96.2296	2.49837	newly 3 weeks cumul	28249.	10147.1	{1182., 20045.}	78.889	0.792975	newly 5 weeks spec	14245.	174.97	{0., 107.}	98.7717	3.44127	newly 5 weeks cumul	58206.	10613.5	{1261., 20059.}	84.0797	1.05859	newly 61 weeks cumul	94140.	10860.3	{1279., 20059.}	88.4636	1.25363
